# Treating male partners of women with bacterial vaginosis (StepUp): a protocol for a randomised controlled trial to assess the clinical effectiveness of male partner treatment for reducing the risk of BV recurrence

**DOI:** 10.1186/s12879-020-05563-w

**Published:** 2020-11-11

**Authors:** Lenka A. Vodstrcil, Erica L. Plummer, Michelle Doyle, Christopher K. Fairley, Colette McGuiness, Deborah Bateson, Jane S. Hocking, Matthew G. Law, Kathy Petoumenos, Basil Donovan, Eric P. F. Chow, Catriona S. Bradshaw, on behalf of the StepUp RCT Team, John Kaldor, Anna McNulty, David A. Lewis, Marcus Y. Chen, Jade E. Bilardi, Sepehr N. Tabrizi, Gerald L. Murray, Jennifer Danielewski, Suzanne M. Garland, Kathy McNamee

**Affiliations:** 1 Central Clinical School, Monash University, Carlton, VIC 3053 Australia; 2 Melbourne Sexual Health Centre, Alfred Health, Carlton, VIC 3053 Australia; 3 Family Planning New South Wales, Ashfield, 2131 Australia; 4 Discipline of Obstetrics, Gynaecology and Neonatology, University of Sydney, Camperdown, 2006 Australia; 5 Centre for Epidemiology and Biostatistics, Melbourne School of Population & Global Health, University of Melbourne, Parkville, 3010 Australia; 6 The Kirby Institute, University of New South Wales, Sydney, 2052 Australia

**Keywords:** Bacterial vaginosis, Antibiotic treatment, Couple treatment, Recurrence, Metronidazole, Clindamycin, StepUp

## Abstract

**Background:**

Bacterial vaginosis (BV) is estimated to affect 1 in 3 women globally and is associated with obstetric and gynaecological sequelae. Current recommended therapies have good short-term efficacy but 1 in 2 women experience BV recurrence within 6 months of treatment. Evidence of male carriage of BV-organisms suggests that male partners may be reinfecting women with BV-associated bacteria (henceforth referred to as BV-organisms) and impacting on the efficacy of treatment approaches solely directed to women. This trial aims to determine the effect of concurrent male partner treatment for preventing BV recurrence compared to current standard of care.

**Methods:**

StepUp is an open-label, multicentre, parallel group randomised controlled trial for women diagnosed with BV and their male partner. Women with clinical-BV defined using current gold standard diagnosis methods (≥3 Amsel criteria and Nugent score (NS) = 4–10) and with a regular male partner will be assessed for eligibility, and couples will then be consented. All women will be prescribed oral metronidazole 400 mg twice daily (BID) for 7 days, or if contraindicated, a 7-day regimen of topical vaginal 2% clindamycin. Couples will be randomised 1:1 to either current standard of care (female treatment only), or female treatment and concurrent male partner treatment (7 days of combined antibiotics - oral metronidazole tablets 400 mg BID and 2% clindamycin cream applied topically to the glans penis and upper shaft [under the foreskin if uncircumcised] BID). Couples will be followed for up to 12 weeks to assess BV status in women, and assess the adherence, tolerability and acceptability of male partner treatment. The primary outcome is BV recurrence defined as ≥3 Amsel criteria and NS = 4–10 within 12 weeks of enrolment. The estimated sample size is 342 couples, to detect a 40% reduction in BV recurrence rates from 40% in the control group to 24% in the intervention group within 12 weeks.

**Discussion:**

Current treatments directed solely to women result in unacceptably high rates of BV recurrence. If proven to be effective the findings from this trial will directly inform the development of new treatment strategies to impact on BV recurrence.

**Trial registration:**

The trial was prospectively registered on 12 February 2019 on the Australian and New Zealand Clinical Trial Registry (ACTRN12619000196145, Universal Trial Number: U1111–1228-0106, https://www.anzctr.org.au/Trial/Registration/TrialReview.aspx?id=376883&isReview=true).

## Background and rationale

Bacterial vaginosis (BV) affects 1 in 3 women globally [1] and increases the risk of important gynaecological and obstetric sequelae including preterm delivery, spontaneous abortion STI and HIV acquisition [2–5]. The global economic burden of treating BV is estimated at USD$4.8 billion annually, with an even greater burden if associated sequelae are included [1]. Current recommended first line treatment for BV is with metronidazole or clindamycin [6], delivered as oral or vaginal therapies for 7 days, with equivalent 1-month BV cure rates of 80% [7], but only 50% of women have been shown to maintain an optimal vaginal microbiota (VM) 3–6 months post-treatment [8]. More effective treatment strategies are needed to improve BV cure, restore and sustain an optimal VM and to reduce adverse health outcomes globally [9].

Evidence that sexual transmission contributes to the pathogenesis of BV has been reported since the 1950s, and led to six male partner treatment trials in the 1980s and 90s [10, 11]. Treatment regimens for males in these trials differed and included oral metronidazole (2 g single dose, 2 g on days 1 and 3 or 500 mg twice daily [BID] 7 days), single dose oral tinidazole (2 g), or oral clindamycin (150 mg four times a day [QID] 7 days) [12–17], with a number of non-standard regimens with respect to current practice. Women were treated with similar regimens to their partner. Only one trial demonstrated a significant impact of partner treatment on recurrence (men received oral metronidazole 2 g single dose vs placebo) [14], and as a consequence, partner treatment was not adopted in clinical practice [10, 18, 19]. However, reviews of the early partner treatment trials also highlighted the significant issues that render their findings inconclusive [10, 18, 19]. Since the publication of these partner-treatment trials, an increasing body of epidemiological data shows women with a regular partner are more likely to experience post-treatment recurrence compared to women with no partner or a new post-treatment partner [8, 20, 21], and microbiological data supports transmission or exchange of BV-associated bacteria (henceforth referred to as BV-organisms) during sexual activity [22–27]. Notably, BV-organisms are more prevalent in the sub-preputial space (under the foreskin) and distal urethra of male partners of females with BV than without BV [22, 26], and recently, the composition of the meatal microbiota was shown to be particularly predictive of incident BV in female partners [24]. A biofilm dominated by BV-organisms has also been detected in male urine and semen more commonly in male partners of females with BV than without BV [28]. Both sexual activity and male circumcision have been shown to influence the penile microbiota [23, 29, 30], with male circumcision prospectively associated with a significant reduction in BV-organisms and a reduction of BV in their female partners [30–32]. Women with a circumcised partner can still develop BV [33] indicating that the sub-preputial space is not the only reservoir of BV-organisms. Collectively this body of evidence suggests that carriage of BV-organisms at both urethral and penile skin sites in men is providing a reservoir from which BV-organisms can be reintroduced to treated women rendering current female treatment strategies inadequate.

The optimal approach to promote clearance of BV-organisms from male partners is, however, unknown. We hypothesise that in order to promote clearance of BV-organisms from both anatomical sites in males, a combination of oral and topical antibiotic therapy may be required. Metronidazole is a nitroimidazole antibiotic with activity against anaerobic bacteria including several BV-organisms such as *G. vaginalis*, *Prevotella* spp., *Bacteroides* spp., *Porphymonas* spp., and *Fusobacterium* spp. [34, 35]. Clindamycin is a lincosamide antibiotic with broad spectrum activity against Gram-positive cocci as well as anaerobic Gram-positive and Gram-negative bacteria [36]. We propose that BV-organisms in the distal urethra of males may be best targeted by oral antibiotics such as metronidazole, while those in the sub-preputial space and coronal sulcus may be most effectively cleared by a topical antibiotic, such as clindamycin cream, which is more suited than a gel to cutaneous application. The additional of topical therapy is also likely to be particularly important in uncircumcised males who have high abundance of sub-preputial BV-organisms [22, 23, 30, 32]. It is possible that the failure of prior male partner treatment trials to include topical antimicrobial therapy contributed to their lack of benefit in women.

### Objectives

In this publication we describe the protocol of a randomised controlled trial (RCT) with the aim to reduce BV recurrence in the female. The primary objective is to determine whether the concurrent treatment of male partners with combined oral metronidazole and topical clindamycin cream (*Intervention group*) reduces the risk of BV recurrence compared to current standard of care, female treatment only (*Control group*), within 12 weeks of enrolment. We hypothesise that male partners who are concurrently treated with antibiotics at the same time as their partner will be less likely to re-infect them with BV-organisms, and their partner will be less likely to experience recurrence within 12 weeks of enrolment compared to women undergoing female treatment only.

### Trial design

This is an open-label, multicentre, parallel-group RCT. Couples assessed for eligibility and recruited will be randomised 1:1 to either the *Intervention group* or *Control group* as described above.

The Standard Protocol Items: Recommendations for Interventional Trials (SPIRIT) checklist is provided as Additional file 1.

## Methods

### Study setting

**Fig. 1:**
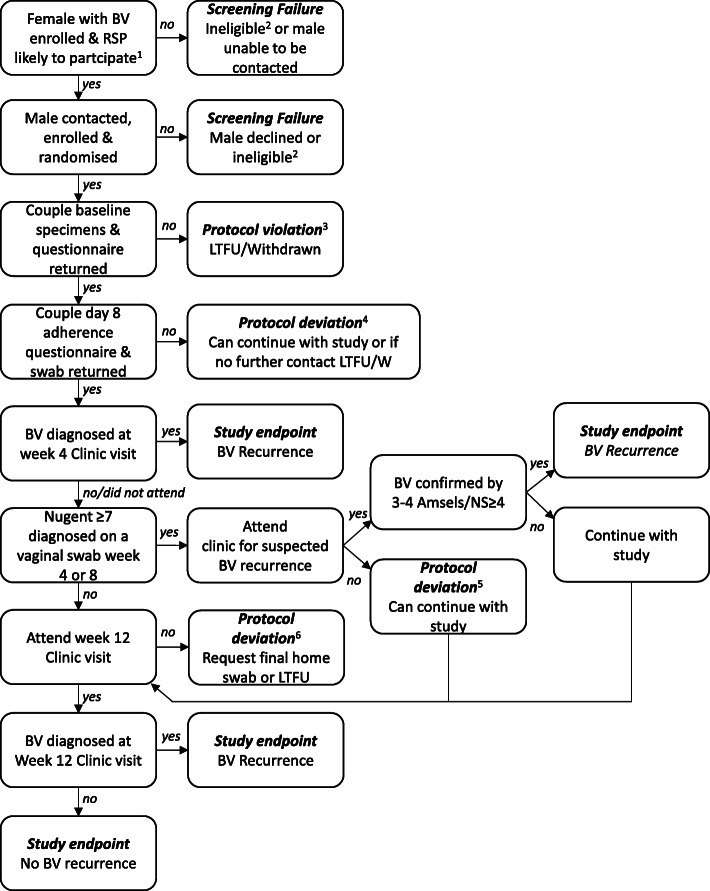
Participant flow through the study from recruitment to study endpoint. ^1^been together ≥8 weeks, indicated/confirmed on phone that RSP likely to participate; ^2^refer to eligibility criteria; ^3^protocol violation means not in modified intention-to-treat (mITT) primary analysis or per protocol analysis; ^4^eligible for mITT and per protocol analyses if week 4 and/or 12 provided but with missing data, not eligible for safety analysis; ^5^if female participant does not attend clinic visits but does return a swab, eligible for secondary endpoints; ^6^if female attends at week 4 but not week 12, still be eligible for mITT; if no visits but returns a swab, eligible for secondary endpoints; if no visits or swabs, protocol violation. Abbreviations: LTFU, Loss to follow up; W, withdrawn

Women will be recruited in three Australian states from sexual health centres (the Central Co-ordinating site, Melbourne Sexual Health Centre [MSHC], Victoria, and Sydney Sexual Health Centre [SSHC], New South Wales [NSW]), and Family Planning centres (Victoria, NSW, South Australia). Women will also be able to be referred from primary care (General practice, GP) or self-refer to the study if they have seen trial posters or the trial website. All women, regardless of recruitment site, will be assessed for clinical-BV prior to enrolment, as described below. Figure 1 illustrates participant flow throughout the trial.

### Participants

#### BV diagnosis methods

The combination of both ≥3 Amsel criteria and a NS = 4–10 will be used to diagnose BV in this trial. This reflects clinical practice and diagnosis of the symptomatic syndrome of BV. Amsel criteria [37] include: i) presence of a characteristic homogenous vaginal discharge; ii) elevated vaginal pH > 4.5; iii) positive amine test (noticeable fishy odour); iv) ≥20% clue cells (epithelial cells coated by adherent gram-negative rods) on microscopy. The Nugent method scores bacterial morphotypes on vaginal Gram stain [38]: i) NS = 0–3 represents a lactobacillus-dominant “optimal” VM, ii) NS = 4–6 represents an intermediate state with few lactobacilli and increased anaerobes, and iii) NS = 7–10 is classified as Nugent-BV. Combining a NS = 4–10 with at ≥3 Amsel criteria optimises the clinical diagnosis of symptomatic BV and importantly captures Amsel positive women with an intermediate NS. Recruitment of women at sites where microscopy is not available will be based on the presence of three Amsel criteria at the bedside: i) presence of a characteristic homogenous vaginal discharge; ii) elevated vaginal pH > 4.5; and iii) fishy odour. A slide for Gram stain of high-vaginal secretions will be collected from women at these sites and sent to MSHC for Nugent scoring and for visualisation of clue cells, and women with NS = 0–3 will be classified as *Screening failures* (see definitions below).

#### Eligibility criteria

Females will be eligible for recruitment if they fulfil the following criteria: are aged 18 years to pre-menopausal (defined as still menstruating within the last 12 months); *and* have symptomatic BV as defined above; *and* have a regular male partner for the prior 8 weeks who is willing and eligible to participate; *and* are being treated with a first line recommended antimicrobial therapy for BV as defined above; *and* have sufficient English language proficiency to understand the study requirements; *and* provide written informed consent; *and* are willing and able to comply with protocol requirements including clinic visits at 4 and 12 weeks; *and* do not have any of the exclusion criteria listed below.

Males nominated as the regular sexual partner of a woman fulfilling inclusion criteria will be *eligible* if they are aged 18 years or above; *and* enrol within a week of their partner with confirmed BV; *and* have sufficient English language proficiency to understand the study requirements; *and* provide written informed consent; *and* are able to comply with protocol requirements; *and* do not have any of the exclusion criteria or contraindications listed for metronidazole.

Prospective participants will be ineligible if they meet any of the following exclusion criteria: are a current sex worker; *or* have concurrent sexual partners; *or* are known to be HIV positive. *Additionally,* *females* *will be excluded if they:* are being currently treated for pelvic inflammatory disease with 14 days of metronidazole with/without ceftriaxone *or* are not being treated with first line recommended antimicrobial therapies for BV at baseline *or* are pregnant or breast feeding; *or* have hypersensitivity or a contraindication to all first line drugs used to treat BV: nitroimidazoles and clindamycin (note they can be enrolled if they are able to take one of these agents). *Additionally,* *males* *will be excluded if they:* have hypersensitivity or a contraindication to *either* nitroimidazoles or clindamycin; *or* if they have significant fungal balanitis.

#### Contraindications

In addition to exclusion criteria above, participants should not be prescribed metronidazole if they have one of the following contraindications: cannot abstain from alcohol during treatment; *or* have a history of epilepsy or central nervous system disorders that predispose to seizures; *or* are known to have peripheral neuropathy; *or* are known to have severe liver disease; *or* are known to have a prolonged QT interval on ECG; *or* are known to have a low neutrophil count; *or* are currently taking warfarin, lithium, disulfiram, phenytoin, phenobarbitone, busulphan, or cimetidine.

### Intervention

All female participants will receive oral metronidazole 400 mg twice daily for 7 days, or if contraindicated, a 7-day regimen of topical vaginal 2% clindamycin cream.

#### Intervention – concurrent male partner treatment

Male partners randomised to the *Intervention Group* will receive concurrent male partner treatment of combined oral metronidazole 400 mg BID and topical 2% clindamycin cream to be applied to the glans penis and upper shaft (under the foreskin if uncircumcised) BID for 7 days.

#### Control – female treatment only (current standard of care)

Male partners randomised to the *Control Group* will not receive treatment and the control for the trial is the current standard of female treatment only. The twice daily application of a topical placebo for 7 days to the penile skin would be highly likely to influence bacterial composition/microbiota. Due to the concerns of this impact on the effect estimate a decision was made against providing a topical placebo to males randomised to the control group.

Adherence will be measured via questionnaire the day following antibiotic therapy (day 8). Participants can discontinue trial treatment and remain in the trial. They can also withdraw from the follow-up assessments at any time.

### Outcome measures

The primary outcome is BV recurrence using current gold standard diagnostic methods defined as ≥3 Amsel criteria and Nugent score (NS) = 4–10 within 12 weeks of enrolment. The secondary outcomes are i) BV recurrence defined as NS ≥ 7 within 12 weeks, ii) BV recurrence defined as ≥3 Amsel criteria within 4 weeks, iii) BV recurrence defined as NS ≥ 7 within 4 weeks, iv) recurrence of a microbiota dominated by BV-associated bacteria within 12 weeks, and v) recurrence of a microbiota dominated by BV-associated bacteria within 4 weeks. All Amsel criteria endpoints require a clinic visit whereas Nugent endpoints can be evaluated using specimens collected at home (see Table 1). Additional measured outcomes include i) adherence to antibiotic therapy (by both women and men), ii) acceptability of antibiotic therapy to men, and iii) adverse effects (AEs) in men (safety analysis).

**Table 1:**
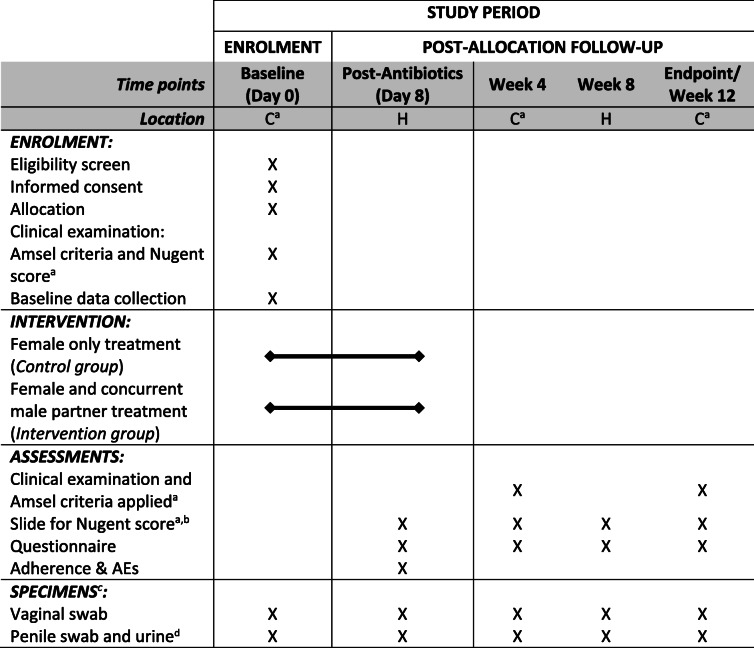
Schedule of enrolment, intervention, assessments and specimen collection

Key: C = clinic visit - required for females only, men may complete at home, H = home procedures; AEs = adverse effects. ^a^required for females only, ^b^a slide containing vaginal secretions is collected for Nugent score; ^c^specimens added to preservative and stored for future molecular studies, ^d^male sample collection at home will be aligned with female samples

### Sample size

BV recurrence rates after recommended therapy are high. Sobel et al reported 50% recurrence within 3 months, and our group reported 52% recurrence within 6 months [8, 39]. We have chosen 12 weeks of follow up, as we believe this will be the optimal duration over which to measure the direct effect of treatment of the male partner on BV recurrence and minimises the risk of introduction of new untreated partners into the “treated partnership”. To obtain an estimate of the efficacy that we could expect from male partner treatment, we have used the reduction in risk reported from consistent condom use (50%) [8, 20] and male circumcision (40–60%) [31]. Based on our data we assume a 12-week BV recurrence rate in the control group of 40%, and conservatively estimate that treating male partners will reduce the risk of BV recurrence in females by 40%. This effect size is at the lower end of that reported from consistent condom use and circumcision.

Data indicate that women with an IUD in situ are more likely to experience BV recurrence [40, 41]. We hypothesise this is due to BV-organisms and biofilm persisting on the IUD after antimicrobial therapy. We will therefore limit recruitment of women with an IUD to no more than 20% of the total study population (68 women/couples), and this will be reviewed at the interim analysis.

With a total sample size of 290 heterosexual couples (145 per arm), we will have 80% power to detect a 40% reduction in BV recurrence from 40% in the control group to 24% in the intervention group (2-alpha = 5%). Assuming a 12-week loss to follow up of 15% in keeping with our previous trials, 342 couples will be recruited (171 couples per arm) to allow for a well-powered primary analysis.

### Participant timeline

#### Recruitment of women with BV

All prospective female participants will first be assessed for BV by a clinician at one of the trial sites. For sites with an onsite laboratory, diagnosis of BV using both Amsel and Nugent criteria will be conducted onsite. For clinicians at one of the sites without an onsite laboratory, a “diagnostic kit” will be provided to standardise clinical diagnosis. The “diagnostic kit” includes the provision of MColorpHast™ vaginal pH indicator strips (pH 2.0–9.0; Merck KGaA, Darmstadt, Germany), a “diagnostic checklist form” to document presence or absence of three Amsel criteria (BV discharge, malodour and pH > 4.5), and a swab and slide for Gram stain of vaginal secretions, that is sent for Nugent scoring and identification of clue cells at the central co-ordinating site. For all women with 3/3 Amsel criteria who are recruited at a site without an onsite laboratory, if the vaginal Gram stain is subsequently scored as NS = 0–3, then the woman will be deemed as a *Screening Failure,* as defined below. When BV is confirmed, women will be referred to the research team and undergo screening procedures.

The trial research nurse will assess eligibility, explain full trial procedures and obtain written consent. Prior to commencing medication, all women will complete a baseline questionnaire, self-collect a high vaginal swab and arrange a time for the research team to contact their partner.

#### Recruitment of male partners

After a recruited woman confirms with the research team that her partner is interested in participating in the trial, a time will be made to speak with him (either immediately if he is present in clinic or via a telephone recruitment consultation). The research nurse will first provide a detailed explanation of the trial, check for trial eligibility and any contraindications to medication, with review by a clinician as required prior to randomisation. For all males, once trial and medical eligibility have been documented in an electronic medical record, written informed consent will be obtained. *For a male present in person* this will be written informed consent from the outset. *For a male on the phone*, initial verbal consent will be documented in the electronic medical record, and then written informed consent will be obtained by return of the signed PICF with the baseline recruitment pack. Following consent, randomisation will be performed. If a male is randomised to the *intervention group*, he will be instructed on how to use the medication. All male participants, regardless of randomisation group will be instructed to collect baseline genitourinary samples (urine and penile skin sample) and refrain from any sexual contact for the 7 days that their partner is taking medication (or, while they are taking their medication if randomised to the *Intervention group*). Study material (i.e. specimen collection materials and questionnaires) and medication (if randomised to the *Intervention group)* will be provided in person or will be couriered to the participant’s home.

### Eligibility for study outcomes

The following definitions will be used to determine participant eligibility for study outcomes and analyses (see Fig. 1).

#### Screening failure

Couples who are deemed a *screening failure* will be ineligible for all study outcomes and not form part of the evaluable population for analyses, including the primary modified intention-to-treat (mITT) analysis and per protocol analysis. A *screening failure* occurs if i) a female declines participation to the full trial following screening at a site without an onsite laboratory, ii) a partner declines participation after the female has enrolled, iii) a couple is unable to be contacted for recruitment procedures, iv) the male is ineligible, v) a female has a screening slide subsequently scored as NS = 0–3 when it processed at the Central Co-ordinating site, and vi) female participants requiring or recalled within the first week of enrolment for treatment of PID requiring 14 days of metronidazole +/− ceftriaxone.

#### Protocol violation

The following are defined as protocol violations and are ineligible for the primary mITT analysis and per protocol analysis (as defined below) but may be eligible for other analyses: i) a female who does not return for any clinical assessment, ii) a female who formally withdraws at any point during the study and has either provided no data at all or states previous data cannot be used, iii) a male participant who formally withdraws prior to day 8 (NB. if he withdraws after providing day 8 data on adherence and his partner is happy to continue her data can still be used in all analyses and his data can be used in safety analyses), iv) a male partner does not provide any baseline specimens, questionnaire or signed PICF, v) a female who does not take any antibiotic therapy, or vi) a male partner randomised to the intervention group who does not use any antibiotic therapy.

#### Protocol deviation

The following are considered protocol deviations i) if a male participant does not return their day 8 questionnaire, they are not eligible for the safety analysis which measures AEs, or ii) if a female participant does not attend any clinic visits for clinical assessment but returns ≥1 swab, she is eligible for the Nugent and microbiota secondary endpoints only.

### Allocation

Allocations will be generated by an independent biostatistician, with study investigators blinded to the randomisation sequence. A 1:1 randomisation ratio to standard of care (female treatment only, *Control group*) or female partner treatment with concurrent male partner treatment (*Intervention group*), in blocks of size four-six, will be used with stratification by recruitment site and known risk factors for recurrence: intrauterine device (IUD)-use and circumcision status. After obtaining verbal consent, the research nurse will log into a password-protected online database, REDCap, an electronic data capture tool hosted and managed by Helix (Monash University) [42], to complete screening and eligibility assessments. The randomisation allocation button will only be visible once all the required information has been entered and the couple are deemed eligible. REDCap will then retrieve the next ‘sealed’ allocation from within each recruitment site and either the IUD-user or non-IUD user stratified allocation and then within these two groups, an uncircumcised or circumcised stratified allocation.

If a protocol violation occurs after randomisation (defined above), reasons will be collected and the participant will be deemed either lost to follow up or withdrawal if this is formally what they decide to do. Couples who fail screening will not be included in the evaluable population for analyses and therefore the next “sealed” allocation can be used to recruit an additional couple.

As this is an open-label RCT, both the research nurse performing randomisation and the participants will know which group they are in. However, the microscopists performing Nugent scoring, clue cell reporting and amine testing will be blinded to randomisation group.

### Study components and data collection methods

**Fig. 2:**
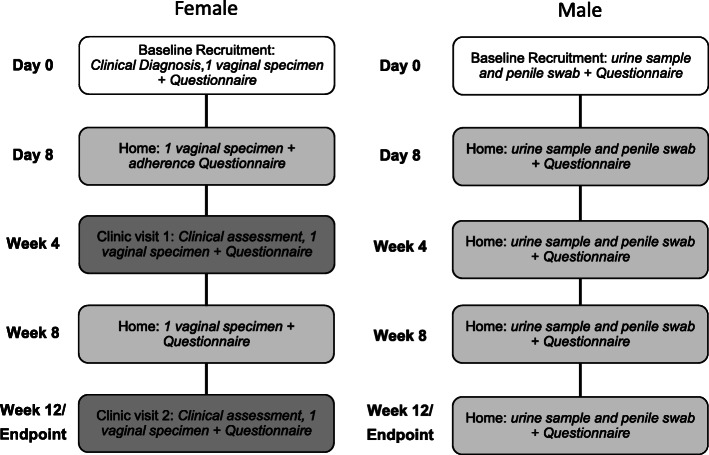
Summary of study schedule and procedures for women and men at each time point

The study constitutes enrolment, day 8 post-antibiotic follow up and then three other follow-up time points. A summary of the study schedule and procedures for couples at each study time point is provided in Table 1 and Fig. 2.

#### Baseline procedures

Once eligibility has been assessed and consent forms signed, participants will be instructed on self-collection of high-vaginal swabs and vaginal secretions on a slide for NS, penile swabs, and urine samples (Table 1, Fig. 2), and provided with a baseline questionnaire. A clinician and pharmacist will instruct participants about antibiotic use and AEs. Couples will be asked to avoid alcohol and to abstain from sex during treatment.

#### Home and clinic follow up

Female participants will collect samples and complete questionnaires on four occasions after baseline including the day after completing antibiotics (day 8), and at weeks 4, 8 and 12 (Table 1, Fig. 2). At scheduled clinic visits (weeks 4 and 12) females will be assessed for BV by Amsel and Nugent criteria, and will complete a questionnaire. At day 8 and week 8, swabs, slides for Nugent scoring, and questionnaires completed at home will be returned by replied paid post to MSHC. If a woman returns a home slide with NS = 7–10, or reports symptoms indicating BV, she will be booked for an interim clinic visit and assessed for BV. All slides collected at home or in non-MSHC clinics will be sent via post to MSHC for scoring within a week. Slides with morphotypes unable to be easily scored will be read by a second independent microscopist and consensus reached.

Male participants will self-sample and complete questionnaires at enrolment and, for those in the *Intervention group*, the day after completing treatment (day 8), or if in the *Control group*, the day after their partner has finished her treatment (Fig. 2). Men will be instructed to complete their follow up specimens and questionnaires at the same time as their partner. All male questionnaires and specimens will be collected at home and returned by replied paid post to MSHC.

Validated questionnaires at each time point will collect data detailing sexual practices (e.g. frequency and types of sex, condom use, change in partnerships), contraceptive use, menstrual dates, genital symptoms, and any interim treatments. Adherence and AEs will be recorded on day 8 questionnaires and participants instructed to call the dedicated study phone if they have any enquiries or are concerned about any AEs experienced.

A reminder SMS will precede each scheduled self-collection time point and reminders sent for up to 2 weeks if samples are not returned, after which a participant will be classified as a non-responder for that interval, but contacted at the next follow-up point. A reminder SMS will be sent to participants 1–2 business days prior to any clinic-based appointment. All couples will receive a $10AUD voucher for each completed stage of follow-up.

#### Study endpoint visit (week 12 or BV recurrence)

The study endpoint is BV recurrence/no BV recurrence within 12 weeks of enrolment. All female participants will be asked to attend a study site for an endpoint clinic visit at week 12, or earlier, if they experience BV recurrence NS = 7–10 on self-collected swab. At the week 12 visit, the research nurse or treating clinician will assess the women for clinical signs of BV using Amsel criteria and take a swab for future molecular studies and slide for Nugent scoring (Table 1) and the participant will be asked to complete the week 12 questionnaire.

#### Treatment of BV recurrence

All women experiencing BV recurrence during the study or at its conclusion will be given an appointment with a clinician and offered treatment accordingly. Couples randomised to the *Control group* will be offered the opportunity for male partner treatment if the female experiences BV recurrence during the study. These data will not be included in the trial but will contribute to a subsequent sub study.

### Collection of genital specimens for future analysis

Genital specimens will be collected at every time point for future molecular analysis (Table 1). Vaginal swabs will be collected using a flocked swab (Copan Diagnostics, Brescia, Italy) and transferred into a vial containing AssayAssure® Multilock (Sierra Molecular, Princeton, NJ, USA) for biological sample stabilisation, preservation and storage. Men will be instructed to retract their foreskin (if applicable) and swab a flocked swab (Copan Diagnostics) pre-moistened with sterile water around the coronal sulcus and over the glans of the penis, prior to transferring into a vial containing AssayAssure® Multilock. In addition, men will be instructed to urinate approximately 20 ml of urine into a standard urine pot. They will be provided with a transfer pipette to then transfer 15 ml into a vial containing urine preserving agent, AssayAssure® Genelock (Sierra Molecular). All specimens will be collected in clinic or sent via post to the Central Co-ordinating site, processed and then stored at -80C for future analysis.

### Statistical methods

Analysis and reporting of the trial will be in accordance with the Consolidated Standard of Reporting Trials (CONSORT) guidelines. A detailed Statistical Analysis Plan will be written close to final data lock, but before any analyses are performed, that will define all analyses in detail. Enrolment and longitudinal specimen and questionnaire data will be entered into two separate password-protected online databases within REDCap and then imported into a statistical package (Stata) for analysis.

The Primary Analysis is a mITT analysis. All randomised women will be analysed as randomised if they 1) returned for ≥1 clinical visit, 2) took one or more doses of antimicrobial therapy, and 3) are part of the evaluable population. Sensitivity analyses will include analyses that take into consideration adherence to treatment (for women and/or men). Women who attend at week 4 but not week 12, are still eligible for the primary outcome analysis. Primary analyses will be simple unadjusted two-group comparisons of the randomised arms using Cox regression. If there are important imbalances in baseline characteristics, further adjusted analyses will be performed. Cox regression analyses will be also used to assess co-variates associated with recurrence including demographics, sexual practices, contraception use, smoking and other factors previously shown to be associated with BV recurrence.

Secondary analyses include a per protocol analysis for participants qualifying for the mITT population and who adhered to ≥70% of doses of the medication(s) prescribed (i.e. ≥10 doses of a 14-dose prescription or ≥ 5 doses of a 7-dose prescription). Any AEs leading to cessation of treatment, and all reported clinical AEs will be summarised descriptively. This will include every male who took ≥1 dose of at least one of the investigational drugs.

#### Planned subgroup analyses

Analyses will be performed for the primary outcome (i.e. no BV recurrence/BV recurrence within 12 weeks of enrolment) comparing the pre-defined randomisation strata: 1) recruitment site type (sexual health clinic or family planning) and the primary outcome, 2) IUD users vs non-IUD users and the primary outcome; and 3) circumcision and no circumcision for the primary outcome. Differences between the randomisation arms within subgroups will only be considered robust if there is statistically significant evidence of an interaction between randomised arm and subgroup.

### Data safety and monitoring

Females receive first line recommended antibiotic therapies, which have been used extensively for decades with an excellent safety profile for the treatment of BV. Metronidazole is also used in a broad range of non-genital anaerobic infections. All female and male participants are assessed for contraindications and drug interactions prior to enrolment. In this study, males are being treated for an indication that is outside approved indications but with Australian Therapeutic Goods Administration (TGA) authorisation (CT-2019-CTN-00046-1). Safety assessments post-treatment will be focused on AEs and symptom-directed assessments. The Trial Management Committee (TMC), consisting the Lead Principal Investigator (PI), co-ordinating PI, and research nurses, will assess whether AEs are thought to be study drug-related and then report relevant cases to the Alfred Hospital Human Research Ethics Committee (HREC) and compile them for the Data Safety Monitoring Board (DSMB), comprising two clinicians with knowledge of sexual health and an independent statistician. The DSMB will review any AEs and advise the Trial Steering Committee (TSC), consisting the lead PI, co-ordinating PI, trial statisticians and epidemiologists, on how to move forward.

#### Planned interim analysis

An interim analysis will be undertaken by an independent researcher not running the trial when the first 150 couples (75 in each arm) have reached the 12-week study endpoint and will include the stated primary and secondary outcome measures of interest. The DSMB will review the interim analysis blinded and a conservative Peto-Prentice stopping rule (*p* < 0.001) will be used to judge whether one arm should be ceased for inferiority only.

#### Trial governance

The TMC will conduct the day-to-day management of the trial, review of protocol deviations, and are responsible for project milestones. The TSC will provide trial oversight and advise on scientific design and rigour. The DSMB, in agreement with the TSC, may suggest changes in the conduct of the study should concerns surrounding the safety of patients arise from review of the interim results or aspects of study conduct that warrant modification (e.g. poor recruitment, IUD-use, substantial losses to follow-up, and concerns regarding safety). The HREC may audit the trial at any time.

#### Confidentiality and dissemination

Any identifying or confidential data will be kept in locked cabinets or password protected computer databases, accessible only by named investigators. All specimens will be de-identified prior to processing. All data will be de-identified and aggregated prior to dissemination in conference abstracts, presentations or publications. Plain language summaries of the findings will be available on the trial site hosted by MSHC at the conclusion of the trial.

## Discussion

The post-treatment recurrence rates for the common vaginal condition, BV, remain unacceptably high. Sex with an ongoing untreated partner has been shown to significantly increase a woman’s risk of post-antibiotic treatment recurrence. A considerable body of evidence now shows that men carry BV-organisms and may be a reservoir for re-infection. This open-label multicentre pragmatic RCT is designed to assess the impact of concurrent male partner treatment with combined oral metronidazole and topical clindamycin with the current standard of care - female treatment only. If effective, concurrent partner treatment will provide an adjunctive therapy for women with a regular partner and may provide the first opportunity for high level sustained BV cure. Additionally, if effective, this approach would also result in improved antibiotic stewardship as women would not require repeated courses of antibiotics for recurrence. This clinical research will be able to be translated to changes to treatment guidelines for BV.

### Trial status

The first participant was recruited on 8 April 2019 and recruitment is expected to continue to 31 December 2022.

## Data Availability

The datasets generated and/or analysed during the current study are not publicly available due to their highly sensitive and confidential nature, but limited data may be available from the corresponding author on reasonable request.

## Abbreviations

AE: Adverse event
BV: Bacterial vaginosis
BID: *Bis in die* (twice daily)
DSMB: Data and Safety Monitoring Board
FP: Family Planning
GP: General Practice
HIV: Human immunodeficiency virus
HREC: Human Research Ethics Committee
IUD: Intra-uterine device
mITT: Modified intention to treat
MSHC: Melbourne Sexual Health Centre
NS: Nugent score
NSW: New South Wales
PI: Principal investigator
QID: *Quarter in die* (four times daily)
RCT: Randomised controlled trial
RSP: Regular sexual partner
STI: Sexually transmitted infection
SSHC: Sydney Sexual Health Centre
TGA: Australian Therapeutic Goods Administration
TMC: Trial Management Committee
TSC: Trial Steering Committee
VM: Vaginal microbiome/microbiota
